# Comparison and Analysis of Biological Agent Category Lists Based On Biosafety and Biodefense

**DOI:** 10.1371/journal.pone.0101163

**Published:** 2014-06-30

**Authors:** Deqiao Tian, Tao Zheng

**Affiliations:** Center for Biosecurity Strategy Management, Beijing Institute of Biotechnology, Beijing, P.R. China; State Key Laboratory of Pathogen and Biosecurity, Beijing Institute of Microbiology and Epidemiology, China

## Abstract

Biological agents pose a serious threat to human health, economic development, social stability and even national security. The classification of biological agents is a basic requirement for both biosafety and biodefense. We compared and analyzed the Biological Agent Laboratory Biosafety Category list and the defining criteria according to the World Health Organization (WHO), the National Institutes of Health (NIH), the European Union (EU) and China. We also compared and analyzed the Biological Agent Biodefense Category list and the defining criteria according to the Centers for Disease Control and Prevention (CDC) of the United States, the EU and Russia. The results show some inconsistencies among or between the two types of category lists and criteria. We suggest that the classification of biological agents based on laboratory biosafety should reduce the number of inconsistencies and contradictions. Developing countries should also produce lists of biological agents to direct their development of biodefense capabilities.To develop a suitable biological agent list should also strengthen international collaboration and cooperation.

## Background

Biological agents include bacteria, viruses, fungi and toxins that cause infection, allergy, toxicity or other hazards to human health, and also pose a serious threat to economic development, social stability and even national security. The classification of biological agents is a basic requirement for biosafety and the development of biodefense capabilities. Biological agent classification can be based on two measures: laboratory biosafety and biodefense considerations. For laboratory biosafety assessment, the main consideration is the ability of biological agents to cause disease and the risk of exposure in laboratory accidents. For biodefense assessment, the main consideration is the potential for biological agent weaponization, terrorism and the harm associated with deliberate release [1]. Biological agents category lists also have other purposes, such as the Select Agents and Toxins List of the United States [2] and the Australia Group list of human and animal pathogens and toxins for export control [3]. However, their principle use is for biosecurity to avoid bioterrorist obtaining or abusing biological agents.

The study and handling of biological agents carries with it the potential for the infection of laboratory personnel and environmental effects. Some countries and organizations have compiled a biological agent category list according to evaluations of the risk of biological agents based on laboratory biosafety. The WHO publishes the “Laboratory Biosafety Manual” [4]. The first edition was published in 1983, second edition in 1993, and the third and latest edition was published in 2004. This manual suggests the criteria for classification of biological agents based on laboratory biosafety, but does not identify the specific category for each biological agent. The National Institutes of Health (NIH) of the United Stated (US) released “NIH guidelines for research involving recombinant DNA molecules” [5], which divided biological agents into four groups, and published the category lists for each biological agent. Since its first publication in June 1994, NIH Guidelines have undergone numerous amendments. The European Union (EU) divided biological agents into four risk groups and in 2000 published the category lists in Directive No. 2000/54/EC “on the protection of workers from risks related to exposure to biological agents at work” [6]. In China “Pathogenic microbiology laboratory biosafety regulations”, which were published by the State Council in 2004 [7], divided pathogenic microorganisms into four groups, with group 1 defined as the highest risk, in contrast to the WHO, NIH and the EU, which defined group 4 as the highest risk. The Chinese Ministry of Health published the “List of human transmission of pathogenic microorganisms” in 2006 [8], which identified harmful levels of each pathogenic microorganism and the laboratory BSL at which they should be handled.

The threat of biological agents not only includes naturally occurring and emerging infectious diseases [9], but also biological weapons and bioterrorism [10]. In 1999, the US Centers for Disease Control and Prevention (CDC) defined and published three groups of potential bioterrorism biological agents in the category list based on assessment of the threat level of biological agents [11], [12]. Following the terrorist attacks of 9.11 and the anthrax mail event in 2001, the European Commission formed a task force on bioterrorism, which became operational in May 2002. As part of its effort, the Commission was tasked with developing lists of agents for which specific activities should be undertaken to improve preparedness in the EU, as a result two groups of potential bioterrorism biological agents were defined [13], [14]. Similarly, Russia evaluated the potential bioterrorism agents and identified three groups of potential bioterrorism agents [15].

In this study, we compared and analyzed the biological agent laboratory biosafety and biodefense category lists and the defining criteria according to specific regions and countries.

## Methods

The Biological Agent Laboratory Biosafety Category Lists and the category criteria of the WHO, NIH, the EU and China as well as the Biological Agents Biodefense Category List and criteria of the CDC, the European Union and Russia were obtained via internet sites and publications. Biological agents included bacteria (*Rickettsia, Chlamydia*), viruses, toxins, fungi and protozoa. Toxins such as ricin, tetrodotoxin, conotoxin, fungi such as *Coccidioides immitis*, and protozoa such as *Cryptosporidium parvum* were included in some category lists, but are not listed in this study.

## Results

### 1. Comparison and analysis of biological agent categorization criteria

Biological agents were divided by the WHO, NIH, EU and China into four laboratory biosafety categories. According to the categorization of the WHO, NIH and the EU, group 1 represents the least risk, while group 4 represents the highest risk. In China, however, categorizations are in reported in the reverse order, with group 4 representing the least risk and group 1 representing the highest risk. In general, the Biological Agent Laboratory Biosafety Category Lists focus mainly on the disease severity, the ability of the agent to spread through the population and whether effective prevention and treatment measures are available. According to the biological agents laboratory biosafety categorization criteria shown in Table 1 (for ease of comparison, the category order of China is reversed), agents in group 1 do not generally cause human disease, while those in group 2 cause disease that is not serious and for which effective treatments and preventive measures are available.Furthermore, the EU and China point out that the risk of transmission of such agents is low. Agents in group 3 cause serious disease. The WHO defines group 3 as the causes of individual infections that are not generally spread, while the EU and China define group 3 as agents that can be spread throughout the population. The NIH, EU define group 3 as agents for which effective treatments and preventive measures are available. Group 4 causes severe disease and is defined by the WHO, NIH and EU as agents for which no effective treatments or preventive measures are available. China includes in this group biological agents that have not been identified in China.

**Table 1:** Comparison of biological agent category criterion based on biosafety.

Group	WHO[4]	NIH[5]	EU[6]	China[7]
1	A microorganism thatis unlikely to causehuman or animaldisease.	Agents that arenot associatedwith disease inhealthy adulthumans.	One that isunlikely to causehuman disease.	Under normalcircumstances, doesnot cause human oranimal disease.
2	A pathogen that cancause human or animaldisease but is unlikelyto be a serious hazardto laboratory workers,the community,livestock or theenvironment. Laboratoryexposure may causeserious infection, buteffective treatment andpreventive measures areavailable and the risk ofspread of infection islimited.	Agents that areassociated withhuman diseasewhich is rarelyserious and forwhich preventiveor therapeuticinterventions areoften available.	One that can causehuman disease andmight be a hazardto workers; it isunlikely to spreadto the community;there is usually effectiveprophylaxisor treatment available.	Can cause human oranimal disease butunder normalcircumstances, it doesnot pose a serioushazard to people,animals or theenvironment, the riskof transmission islimited, Laboratoryinfection rarely causesserious illness witheffective treatment andprevention.
3	A pathogen that usuallycauses serious human oranimal disease but doesnot ordinarily spread fromone infected individual toanother. Effectivetreatment and preventivemeasures are available.	Agents that areassociated withserious or lethalhuman disease forwhich preventiveor therapeuticinterventions maybe available (highindividual risk butlow communityrisk).	One that can causesevere human diseaseand present a serioushazard to workers; itmay present a risk ofspreading to thecommunity, but thereis usually effectiveprophylaxis ortreatment available.	Can cause serioushuman or animaldisease. It is relativelyeasy to spread betweenpeople, animals andpeople, among animals,directly or indirectly.
4	A pathogen that usuallycauses serious human oranimal disease and thatcan be readily transmittedfrom one individual toanother, directly or indirectly.Effective treatment andpreventive measures are notusually available.	Agents that arelikely to causeserious or lethalhuman disease forwhich preventive ortherapeuticinterventions are notusually available(high individual riskand high communityrisk).	One that causes severehuman disease and isa serious hazard toworkers; it may presenta high risk of spreadingto the community; thereis usually no effectiveprophylaxis or treatmentavailable.	Can cause very seriousdisease in human andanimal, includingbiological agents hasnot been found in China

**Note:** For ease of comparison, the category order of China is reversed.

Biological agent biodefense categorization is not as common as laboratory biosafety categorization, mainly having been defined by the US CDC, the EU and Russia. Unlike laboratory biosafety criteria, not all agents are divided into four groups. For biodefense categorization, the US CDC and Russia divided biological agents into three groups, with the EU defining two groups. For biodefense categorization, group 1 represents the highest threat, unlike the WHO, NIH and EU laboratory biosafety category lists, for which group 4 is defined as the highest threat.

The US CDC biological agents biodefense evaluation criteria include: (1) can be easily disseminated or transmitted from person to person; (2) result in high mortality rates and have the potential for major public health impacts; (3) might cause public panic and social disruption; (4) require special action for public health preparedness [12]. EU determine the degree of threat associated with biological agents according to the formula T = (B * M * A * D)-Tr+C, where T is the threat level, B is the base score, M is the mortality rate, A is the aerosol spread of ability, D is the ability to spread between people, Tr is drugs and vaccines for a possible response and C is the production potential. The base score includes the current prevalence in Europe and also refers to the US CDC category list. Ability to spread among people includes the number of susceptible people. Production potential includes the acquisition of potential pathogens, stability, and potential production capacity [14]. Russia divided potential bioterrorism agents into three categories with the main factors to consider including: (1) human sensibility to the microbe; (2) infectious dose for infection via aerosol; (3) contagiousness; (4) possible routes of infection; (5) survival in aerosol and in the environment; (6) characteristics of the disease such as severity, lethality and disease period; (7) possibility of mass production of the bioagent; (8) possibility of rapid diagnosis; (9) various means of prophylaxis; (10) various means of treatment [15].

In comparison with the Biological Agent Biosafety Category Lists, the development of Biological Agent Biodefense Category lists requires the consideration of more factors.The development of Biological Agent Biodefense Category Lists generally takes into account characteristics of individual biological agent, such as their acquisition, production and dissemination capabilities, results of deliberate release and response measures. In addition, the US CDC takes into account the need for special public health response measures, the EU takes into account the epidemiological situation and disease susceptibility factors and Russia takes into account the dose required for aerosol infection.

### 2. Biological agent category list comparison and analysis

Comparison of the biological agent category lists revealed that the laboratory biosafety risk groups of most biological agents in NIH, EU and China are consistent, although some inconsistencies were identified (Table 2, Table 3). *Bacillus anthracis* was included in NIH biosafety category risk group 2, but was included in risk group 3 according to the EU categorization. Venezuelan equine encephalitis virus and Yellow fever virus were included in risk group 1 of the Chinese biosafety categorization (equivalent to the risk group 4 according to the WHO, NIH and EU), but were included in risk group 3 according to NIH and EU categorization. Eastern and Western equine encephalitis viruses were included in risk group 1 of the China biosafety categorization, but were included in risk groups 2 and 3 according to NIH and EU categorization, respectively. Monkeypox virus and St. Louis encephalitis virus were included in risk group 1 of the Chinese biosafety categorization, but were included in risk group 3 in the NIH and EU categories. Kyasanur Forest Virus and Omsk Hemorrhagic fever virus were included in risk group 4 according to NIH biosafety categorization, but were included in risk group 3 by the EU.

**Table 2:** Bacteria (*Rickettsia*, *Chlamydia*) biosafety and biodefense category list comparison.

Biological agents	Biosafety category			Biodefense category		
	NIH	EU	China	US CDC	EU	Russian
*Bacillus anthracis*	2	3	2	Category A	Very high threat	Group 1
*Yersinia pestis* a	3(2)	3	2	Category A	Very high threat	Group 1
*Francisella tularensis* b	3(2)	3(2)	2	Category A	Very high threat	Group 1
*Clostridium botulinum* c	2	2	3	Category A	Very high threat	Group 1
*Burkholderia mallei*	3	3	2	Category B	Very high threat	Group 1
*Burkholderia pseudomallei*	3	3		Category B	High threat	
*Rickettsia prowazekii* d	3	3	2	Category B	High threat	Group 1
*Rickettsia rickettsii* d	3	3	2		High threat	
*Coxiella burnetii* e	3(2)	3	2	Category B	High threat	Group 1
*Brucella species*	3	3	2	Category B	High threat	Group 2
*Staphylococcus aureus* c	2	2	3	Category B		Group 3
*Clostridium perfringens* c		2	3	Category B		
*Vibrio cholerae* f	2	2	2	Category B	High threat	Group 2
*Salmonella species* g	2	3(2)	3	Category B	High threat	Group 3
*Shigella species* h	2	3(2)	3	Category B	High threat	Group 3
*Escherichia coli O157:H7* i	2	3	3	Category B	High threat	
*Chlamydia psittaci* j	2	3(2)	3	Category B	High threat	
*Corynebacterium diphtheriae*	2	2	3		High threat	Group 2
*Legionella pneumophila*	2	2	3		High threat	
*Mycobacterium tuberculosis*	3	3	2		High threat	

a NIH laboratory biosafety categorization: *Yersinia pestis* specifically pgm(–) strains (lacking the 102 kb pigmentation locus) and lcr(–) strains (lacking the LCR plasmid) are in group 2.

b NIH laboratory biosafety categorization: *Francisella tularensis* subspecies novicida, strain Utah 112; *F. tularensis* subspecies *holarctica LVS*; *F tularensis biovar tularensis* strain ATCC 6223 are in group 2. EU laboratory biosafety categorization, *F. tularensis* type A is in group 3, while type B is in group 2.

c Biosecurity categorization: *Clostridium botulinum*, *Staphylococcus aureus* and *Clostridium perfringens* are listed as *Clostridium botulinum* toxin, *Staphylococcal* enterotoxin B, epsilon toxin of *Clostridium perfringens*.

d Chinese laboratory biosafety categorization: *Rickettsia spp* is in group 2.

e NIH laboratory biosafety categorization: *Coxiella burnetii* – specifically the Phase II, Nine Mile strain, plaque purified, clone 4 is in group 2.

f Chinese laboratory biosafety categorization: *Vibrio cholerae* epidemic strains are managed as risk group 2, non-epidemic strains are managed as risk group 3.

g EU laboratory biosafety categorization: *Salmonella typhi* is in group 3, while other salmonella strains are in group 2.

h EU laboratory biosafety categorization: *Shigella dysenteriae* type 1 is in group 3, while other strains are in group 2.

i Chinese laboratory biosafety categorization: pathogenic *Escherichia coli* are listed.

j EU laboratory biosafety categorization: *Chlamydia psittaci* avian strains are in group 3, while other strains are in group 2. “*Chlamydia psittaci”* is now reclassified as “*Chlamydophila psittaci”.*

**Table 3:** Virus biosafety and biodefense category list comparison.

Biological agents	Biosafety category			Biodefense category		
	NIH	EU	China	US CDC	EU	Russian
Variola major^a^		4	1	Category A	Very high threat	Group 1
Ebola virus	4	4	1	Category A	Very high threat	
Marburg virus	4	4	1	Category A	Very high threat	Group 1
Lassa virus	4	4	1	Category A	Very high threat	
Machupo virus	4	4	1	Category A	Very high threat	
Crimean-Congo hemorrhagic fever virus^b^	4	4	1		Very high threat	
Guanarito virus	4	4	1		Very high threat	
Junin virus^c^	4(2)	4	1		Very high threat	
Omsk Hemorrhagic Fever Virus	4	3	1		Very high threat	
Sabia virus	4	4	1		Very high threat	
Venezuelan equine encephalitis^d^	3(2)	3	1	Category B	High threat	
Eastern equine encephalitis	2	3	1	Category B	High threat	
Western equine encephalitis	2	3	1	Category B	High threat	
Influenza virus^e^	3(2)	2	2(3)		High threat	Group 1
Japanese Encephalitis Virus^f^	3(2)	3	2		High threat	Group 2
Yellow fever virus^g^	3(2)	3	1(3)		High threat	Group 2
Rift Valley fever virus^h^	3(2)	3	2		High threat	
Monkey pox	3	3	1		High threat	
Kyasanur Forest Virus	4	3	1		High threat	
St. Louis Encephalitis Virus	3	3	1		High threat	
West Nile Virus	3	3	2		High threat	
Nipah virus			1	Category C	High threat	
SARS- associated coronavirus (SARS-CoV)	3		2			
Hantavirus^i^	3	3(2)	2	Category C	High threat	
Human immunodeficiency virus (HIV)	3	3	2			Group 3
Rabies	2	3	2			Group 3
Dengue virus	2	3	3			

a Smallpox is caused by Variola viruses. Variola viruses including Variola major which causes disease with serious clinical symptoms and Variola minor (alastrim) which causes disease with less severe clinical symptoms. NIH laboratory biosafety categorization:, Variola major is not listed, but Variola, alastrim and whitepox are restricted to a single national facility (World Health Organization Collaborating Center for Smallpox Research, Centers for Disease Control and Prevention, Atlanta, Georgia); EU laboratory biosafety categorization: Variola major and Variola minor are in group 4; Chinese laboratory biosafety categorization: Variola virus and alastrim virus are in group 1.

b EU biodefense categorization: Congo-Crimean hamorrhagic fever virus is listed.

c NIH laboratory biosafety categorization: Junin virus candid #1 vaccine strain is in group 2.

d NIH laboratory biosafety categorization: Venezuelan equine encephalitis is in group 3, Venezuelan equine encephalitis vaccine strains TC-83 and V3526 are in group 2.

e NIH laboratory biosafety categorization: Influenza virus is in group 2, 1918H1N1, human H2N2 (1957–1968) and highly pathogenic avian influenza H5N1 strains are in group 3. EU biodefense categorization: lists influenza virus new strains.

f NIH laboratory biosafety categorization: Japanese encephalitis virus is in group 3, Japanese encephalitis virus vaccine strain SA 14-14-2 is in group 2. EU laboratory biosafety categorization: lists Japanese B encephalitis.

g NIH laboratory biosafety categorization: yellow fever virus vaccine strain 17D is in risk group 2. Chinese laboratory biosafety categorization: yellow fever virus is in group 1, yellow fever virus vaccine strain (17D) is in group 3.

h NIH laboratory biosafety categorization: rift valley fever virus is in group 3, rift valley fever virus vaccine strain (MP-12) is in group 2.

i NIH laboratory biosafety categorization: Hantaviruses including Hantaan virus are in risk group 3. EU laboratory biosafety categorization: Hantaan (Korean hemorrhagic fever) and Seoul virus are in risk group 3, other Hantaviruses are in risk group 2.

Most of the biological agents in the biodefense category lists of the US CDC, EU and Russia were found to be consistent. For example, *Bacillus anthracis*, *Yersinia pestis*, *Francisella tularensis*, *Variola* major and Marburg virus were all listed in the highest threat group. However, some inconsistencies were identified. For example, *Burkholderia mallei* was listed in the highest threat group according to the biodefense categorization of the EU and Russia, while the US CDC listed this agent in the second highest category. *Rickettsia prowazekii*, *Coxiella burnetii* were listed in the highest threat group according to the Russian biodefense categorization, but was included in the second highest category by the US CDC and the EU. Influenza virus was listed in the highest threat group according to the Russian biodefense categorization, but was included in the second highest category by the EU, and was not specifically listed by the US CDC.

In general, the grade order of most biological agents of laboratory biosafety category list and biodefense category lists was found to be consistent. Commonly, agents included in a high laboratory biosafety category (such as *Variola* major, Ebola virus and Marburg virus) were categorized accordingly at the higher biological defense level and those categorized at lower laboratory biosafety levels (such as *Salmonella* and *Shigella*) were also included categorized at lower biodefense levels. However, some inconsistencies were identified, such as *Bacillus anthracis*, *Yersinia pestis*, *Francisella tularensis* which were categorized according to the highest biodefense level, but were not included in the corresponding laboratory biosafety level. Similarly, immunodeficiency virus and *Mycobacterium tuberculosis* were categorized according to the highest laboratory biosafety level, but were not included in the corresponding biodefense level. This inconformity is relative to the different purposes and criteria of the two types of category list.

## Conclusions

Classification of biological agents based on laboratory biosafety facilitates enhanced biological agent management and reduces the incidence of laboratory personnel infections and environmental contamination. The classification of biological agents based on biodefense provides a focus for improving biodefense capabilities, such as biodefense science and technology layouts and assisting in the prioritization of vaccine and therapy development. In defining the biological agent category list and strengthening biosafety and biodefense, the following aspects require consideration.

### 1. Biological agent classification based on laboratory biosafety should reduce inconsistencies and contradictions

The WHO has published biological agent laboratory biosafety risk group classification criteria, but has not published the category list based on this classification. Some countries refer mainly to the biological agent biosafety category lists published by the US CDC, NIH or EU.

The category lists and criteria published by the US CDC, NIH, EU or China have certain inconsistencies. For example, in the WHO laboratory biosafety standard, the third risk group is defined as, “not ordinarily spread from one infected individual to another”, but the EU criteria states that, “it may present a risk of spreading to the community”. According to the WHO standard, the NIH and EU list should not include *Mycobacterium tuberculosis*, SARS-CoV and other group 3 biological agents.

In addition, the names of biological agents are occasionally inconsistent. The EU and NIH laboratory biosafety categorization lists the Equine Morbilli Virus, while the same virus is designated as Hendra virus in the Chinese laboratory biosafety category list. These inconsistencies could impede international communication and delay situation management.

### 2. Developing countries should also have biological agent biodefense lists to direct the development of biodefense capabilities

Bioterrorism and biowarfare poses an enormous threat to humankind. Many biological agents have no effective preventive or treatment measures, and even usable vaccines and drugs often have serious side effects. Therefore, the development of more effective and safer prophylactic and therapeutic measures is urgently required. The United States has launched “Project Bioshield” and other project to strengthen their biological defense capability [16], which is mainly based on the CDC biological agent list. The EU and countries in other regions also attach great importance to the development of biological defense capabilities, based on their respective biological agent lists.

However, bioterrorism and biowarfare threats are not unique to developed countries. Some developing countries are densely populous, with scant biodefense budgets, and bioterrorism would be even more effective in low resourced regions. Developing countries should also have biodefense biological agent lists. Some countries can refer to the biological agent list of US CDC, but this is not a universal list as it may be suitable for the United States. During the developments of Biological Agent Biodefense Lists, developing countries should evaluate characteristics of each biological agent and threats faced, existing biodefense capabilities based on its specific regional conditions. The CDCs of developing country should play an important role in this process and cooperate with other related departments and organizations.

### 3. The development and revision of biological agent lists requires international cooperation and collaboration

From the differences in the biological agent category list based on laboratory safety or biodefense, it can been seen that worldwide cooperation and collaboration is lacking, the criteria for inclusion on laboratory biosafety lists are not uniform, the names of some biological agents differ between countries, and the list orders are contradictory. In the making and revising of biological agent lists, international level discussion is highly important.

With regards to biodefense, international cooperation is even more absent. Biodefense is often considered as a sensitive field, and it may be simpler to collaborate well in other scientific areas such as cancer or HIV research. Lessons from such fields could be applied to the area of biodefense collaboration.

The development of laboratory biosafety and biodefense capabilities require shared experience to face the pressing questions of modern-day threat. Cooperation can benefit each other, including further discussions into the making or revision of biological agent category lists.
